# Longitudinal Associations Between Food Insecurity and Suicidal Ideation Among Adults Aged ≥65 in the Korean Welfare Panel Study

**DOI:** 10.3389/ijph.2023.1605618

**Published:** 2023-06-05

**Authors:** Jeongyoon Lee, Tae-Young Pak

**Affiliations:** Department of Consumer Science and Convergence Program for Social Innovation, Sungkyunkwan University, Seoul, Republic of Korea

**Keywords:** suicidal ideation, food insecurity, income support, meal delivery, seniors

## Abstract

**Objectives** *:* This study aimed to explore longitudinal associations between food insecurity and suicidal ideation, and the moderating roles of intervention programs.

**Methods:** Data were derived from the 2012–2019 waves of the Korean Welfare Panel Study. Participants aged ≥ 65 at baseline (*n* = 4,425) and their annual follow-up measurements for a mean of 6.58 years were included. Conditional fixed effects logistic regressions were used to test 1) associations between food insecurity and the onset of suicidal ideation; 2); whether associations were attenuated by food assistance and income support programs.

**Results:** Food insecurity was associated with higher odds of suicidal ideation in the full sample (OR, 1.77; 95% CI, 1.37–2.29), among women (OR, 1.67; 95% CI, 1.24–2.26) and men (OR, 2.06; 95% CI, 1.25–3.40). The association between food insecurity and suicidal ideation was attenuated by participation in home-delivered meal services (OR, 0.43; 95% CI, 0.21–0.88).

**Conclusion:** Food insecure older adults were more likely to consider committing suicide than their food secure counterparts. Food assistance through home-delivered meal services, but not other intervention programs, could weaken this link.

## Introduction

Suicide rates in South Korea (Korea, hereafter) show a significant age-related pattern, with a disproportionately greater number of older adults committing suicide. Among the 13,799 suicides that occurred in 2019, 35.1 percent were persons aged 60 or older [1]–a group that represents only one-fifth of the country’s population. Suicidal risk among older adults is known to be affected by biological and health characteristics, as well as by environmental and contextual factors, including socioeconomic status [2–4]. Additionally, the etiology of suicide from ideation (having thoughts, ideas, or ruminations about the possibility of ending one’s life) to planning, attempt, and completion is significantly associated with disadvantaged economic status and stressful events that occur at older ages [5].

Food insecurity is defined as “limited or uncertain availability of nutritionally adequate and safe foods, or limited or uncertain ability to acquire food in socially acceptable ways” [6]. It is a multi-faceted concept that measures both the limited access to sufficient amounts of food and inability to meet dietary needs and preferences to lead a healthy life. Research on older adults in Korea indicates that, approximately one in ten experiences food insecurity, which is notably higher than the national average across all age demographics [7]. Food insecurity tends to co-occur with poverty and socioeconomic adversity, which could undermine the health and wellbeing of older adults. The existing evidence shows that food insecurity is independently associated with insufficient nutrition intake [8] and diet-related chronic conditions, such as cardiovascular diseases, hypertension, and type 2 diabetes [9, 10].

There is a paucity of research examining the relationships between food insecurity and suicide among older adults. The pathway between food insecurity and suicide is suggested by a series of studies, linking senior hunger to mental health issues, including anxiety, stigmatization, psychological distress, and depressive symptomology [11–16]. More direct evidence on food insecurity and increased risk of suicide has been reported by the cross-sectional studies of older adults in the US [17] and the six developing countries [18]. Food insecurity is a particularly relevant risk factor of elderly suicide in Korea, as nearly 50 percent of older adults suffer from relative poverty [19]. The growing elderly population in Korea has spurred concerns that food insecurity will worsen in this age group, and a greater number of low-income seniors will consider suicide as an exit from hunger [20]. Thus, it is important to examine the association between food insecurity and suicidal ideation among Korean older adults, and explore possible interventions that could alleviate this situation.

Food insecure seniors in Korea receive nutrition support from the food assistance programs dedicated to offering prepared and packaged meals or use cash benefits from the minimum income support programs to supplement their food-related budgets. The congregate meal program distributes nutritious meals in a group setting through designated community centers or charitable organizations. Older adults with limited mobility receive their packaged meal boxes at home, using the home-delivered meal services. The National Basic Livelihood Security program provides cash assistance to households living below the poverty line. The Basic Pension program provides pension coverage to older persons ≥ 65 who are in the bottom 70 percent of the income distribution in the country [21]. Studies regarding older Americans have proved that food assistance programs like the Supplemental Nutrition Assistance Program (SNAP) mitigate the adverse health consequences of food insecurity [12, 15, 22]. However, whether the current nutrition assistance programs in Korea yield similar health benefits remains underexplored in the literature.

This study uses longitudinal data from community-dwelling residents in Korea to examine the association between food insecurity and suicidal ideation in older adults and the potential moderating roles of government interventions. This study addressed the gap in the literature by providing longitudinal evidence regarding the food insecurity-suicidality link, and examining efficacy of intervention programs. Cross-sectional studies have reported an increased risk of suicide among food-insecure older adults [3, 17, 18], but none have demonstrated this association in a longitudinal study setting. Using time-varying components of data allows for the observation of long-term associations between the variable of interest and outcomes, and uncover individual patterns of change before and after exposure (e.g., provision of food assistance). In this study, we hypothesize that a) changes in food insecurity are associated with changes in suicidal ideation over time, and b) food assistance and income support programs moderate this longitudinal association.

## Methods

### Study Sample

The study sample was obtained from the 2012–2019 waves of the Korean Welfare Panel Study (KoWePS). The KoWePS is an ongoing longitudinal study covering health and social welfare aspects of a nationally representative Korean households, conducted by the Korean Institute of Social and Health Affairs in conjunction with the Social Welfare Research Institute of Seoul National University. The study began in 2006 and has been conducted annually since 2006. The initial sample comprised 18,856 respondents from 7,072 households, selected by a two-stage stratified cluster sampling method. Data were collected through face-to-face interviews held at participants’ households, using structured questionnaires.

The study sample is restricted to respondents who were aged 65 or older in 2012. Respondents who did not participate in the follow-up surveys were excluded from the data so that the regression models exploit within-variation in the survey responses. After listwise deletion of missing data, a total of 29,138 person-year observations from 4,425 baseline participants (3,409 households) were retained in the sample.

### Food Insecurity

Food insecurity was measured using the six-item short form of the Korean Household Food Security Survey (KHFSS). The KHFSS is an 18-item food insecurity scale based on the United States Department of Agriculture’s (USDA) Food Security Scale [6]. The measurement items were designed to capture household experiences of food hardships in the past year, including anxiety about food insufficiency and reduced food intake or dietary changes caused by financial constraints (Supplementary Table S1). Each item was assigned a score of 1 if a respondent offered an affirmative response (often/sometimes, yes, or almost every month/some months, but not every month) and 0 otherwise. The summed score ranges from 0 to 6, indicating the degree of food insecurity. Households were classified into food secure (total score ≤1) or food insecure (total score ≥ 2) groups, based on the USDA-specified threshold for food insecurity [6]. This study does not distinguish moderate and severe food insecurity, because the two-level categorization results in sparse data for some cross-tabulated cells.

### Suicidal Ideation

Suicidal ideation was based on an affirmative response to the survey question “Have you had any serious thoughts regarding suicide in the preceding year?” Participants were categorized as having had suicidal ideation (coded = yes) or not (coded = no). Other response options (i.e., do not know or refusal) were treated as missing data, and excluded from the sample.

### Food Assistance and Income Support Programs

Government support variables included two measures of food assistance programs (congregate meal program, home-delivered meal services) and two measures of income support programs (National Basic Livelihood Security, Basic Pension). Participants were asked, “Have you used the following government service in the preceding year?”, and provided with a list of welfare programs including the aforementioned food assistance and income support programs. They were instructed to tick yes or no on the program they had participated, and the responses were binary coded to construct indicators of program participation.

### Covariates

Regression models were adjusted for potential confounders that could affect the associations between food insecurity and suicidal ideation. The covariates included age, marital status, number of household members, self-rated health, chronic diseases, smoking, number of private health insurance coverages, employment status, household income (in quintiles), and region of residence. Age was measured in continuous terms, subtracting the year of birth from the survey year. Marital status was categorized as currently married or not in marital relationship (separated, divorced, widowed, never married). Self-rated health was measured by asking respondents to rate their overall health on a 5-point Likert scale, ranging from very poor to very good. Chronic diseases were coded as one for respondents who had chronic condition(s) for 6 months or more. Employment status was recoded as employed, self-employed, or retired. Household income was self-reported by the householders in continuous terms and collapsed into quintiles.

### Statistical Analysis

Conditional fixed effects (FE) logistic regression [23] was used to evaluate the extent to which food insecurity is associated with the risk of suicidal ideation, after controlling for potential confounders. The FE regression uses subject-demeaned data to estimate the degree of association between changes in independent and dependent variables. As the FE regression utilizes variation within a subject over time, factors that do not change between waves are omitted from the regressions. The FE regression has advantages over cross-sectional regressions as it controls for the confounding effects of unobserved time-invariant factors and reveals dynamic relationships between exposure variables and outcomes.

In alignment with our research questions, we designed two sets of regression models: a) a conditional FE logistic regression of suicidal ideation on food insecurity and covariates, and b) a conditional FE logistic regression of suicidal ideation on food insecurity, government support indicators, their interactions, and covariates. The logistic regressions were further weighted by inverse probability weights (IPW), where the weights are 1/PS for the food insecure respondents and 1/(1-PS) for the food secure respondents. The IPW method approximates a random exposure to food insecurity and removes confounding by creating a pseudo population in which the suicidal outcome is independent of the covariates. For all logistic regressions, adjusted odds ratios were reported along with 95% confidence intervals. We conducted two-sided hypothesis testing, considering coefficients with *p*-values less than 0.05 as statistically significant. All analytical procedures were performed using Stata version 16.1 (StataCorp LLC, College Station, TX).

## Results

Table 1 presents baseline characteristics of 4,425 participants at their first response by suicidal ideation and food insecurity categories. The study sample is 62% female (38% male), 59% married (41% separated, divorced, widowed, or never married), 71% retired (10% employed; 19% self-employed), and the mean age is 73.67 years. About 5% of the participants were food insecure, and 4% reported instances of suicidal ideation in the preceding year.

**TABLE 1:** Baseline distribution of the study sample by suicidal ideation and food insecurity (Korean Welfare Panel Study, South Korea, 2012–2019).

Variables	By suicidal ideation		By food insecurity		All (*n* = 4,425)
	Suicidal ideation (*n* = 208)	No suicidal ideation (*n* = 4,217)	Food insecurity (*n* = 181)	Food secure (*n* = 4,244)	
Suicidal ideation			0.13	0.04	0.05
Food insecurity	0.12	0.04			0.04
Age (yrs)	73.00	73.71	74.67	73.63	73.67
Sex					
Female	0.66	0.62	0.75	0.61	0.62
Male	0.34	0.38	0.25	0.39	0.38
Marital status					
Currently married	0.52	0.59	0.30	0.60	0.59
Not in marital relationship	0.48	0.41	0.70	0.40	0.41
Number of household members	1.77	2.06	1.62	2.07	2.05
Self-rated health					
Very good	0.01	0.02	0.01	0.02	0.02
Good	0.11	0.24	0.12	0.24	0.23
Fair	0.24	0.32	0.25	0.31	0.31
Poor	0.58	0.40	0.55	0.40	0.41
Very poor	0.06	0.03	0.07	0.03	0.03
Chronic conditions					
Six months or more	0.89	0.85	0.86	0.85	0.85
Less than 6 months	0.11	0.15	0.14	0.15	0.15
Smoking					
Currently smoking	0.20	0.11	0.12	0.11	0.11
Not currently smoking	0.80	0.89	0.88	0.89	0.89
Private health insurance plans	0.11	0.18	0.06	0.18	0.18
Employment status					
Employed	0.10	0.10	0.09	0.10	0.10
Self employed	0.07	0.20	0.10	0.19	0.19
Retired	0.84	0.71	0.81	0.71	0.71
Household income					
Quintile 1	0.75	0.51	0.87	0.51	0.52
Quintile 2	0.14	0.29	0.12	0.29	0.28
Quintile 3	0.06	0.12	0.01	0.12	0.11
Quintile 4	0.02	0.06	0.01	0.06	0.05
Quintile 5	0.02	0.03	0.01	0.03	0.03
National Basic Livelihood Security					
Received	0.30	0.11	0.41	0.11	0.12
Not received	0.70	0.89	0.59	0.89	0.88
Basic Pension					
Received	0.87	0.75	0.93	0.75	0.76
Not received	0.13	0.25	0.07	0.25	0.24
Free meal services					
Received	0.14	0.18	0.18	0.18	0.18
Not received	0.86	0.82	0.82	0.82	0.82
Home-delivered meal services					
Received	0.10	0.02	0.12	0.02	0.02
Not received	0.90	0.98	0.88	0.98	0.98
Region of residence					
Metropolitan city	0.42	0.34	0.37	0.34	0.35
Urban	0.33	0.32	0.39	0.32	0.33
Rural	0.25	0.33	0.24	0.33	0.33

Table 2 reports the association between food insecurity and suicidal ideation estimated by conditional FE logistic regression. The baseline model shows that food insecurity is associated with 2.00 (95% CI, 1.56–2.57) times higher odds of suicidal ideation, conditional on region of residence. The association remained statistically significant when the model was further adjusted for demographic factors (OR, 1.80; 95% CI, 1.40–2.33) and demographic and socioeconomic factors (OR, 1.77; 95% CI, 1.37–2.29). The fully adjusted model, conditional on demographic, socioeconomic, and government support indicators, showed an odds ratio of 1.77 for suicidal ideation (95% CI, 1.37–2.29). Balancing out the covariate heterogeneity between food secure and insecure participants leads to odds ratio of 2.36 for suicidal ideation (95% CI, 1.77–3.15). In the subsample analysis by gender, we find that the estimated odds ratios are more pronounced for men, relative to women, across model specifications.

**TABLE 2:** Association of food insecurity with suicidal ideation, conditional fixed effects logistic regression results (Korean Welfare Panel Study, South Korea, 2012–2019).

	Predictor	Full sample		Women		Men	
		OR (95% CI)	*p*-value	OR (95% CI)	*p*-value	OR (95% CI)	*p*-value
Baseline^a^	Food insecurity	2.00 (1.56, 2.57)	<0.001	1.93 (1.44, 2.58)	<0.001	2.26 (1.40, 3.66)	0.001
Demographic^b^	Food insecurity	1.80 (1.40, 2.33)	<0.001	1.69 (1.25, 2.27)	0.001	2.16 (1.32, 3.53)	0.002
Demographic and SES^c^	Food insecurity	1.77 (1.37, 2.29)	<0.001	1.67 (1.24, 2.25)	0.001	2.05 (1.25, 3.36)	0.004
Demographic, SES, and welfare^d^	Food insecurity	1.77 (1.37, 2.29)	<0.001	1.67 (1.24, 2.26)	0.001	2.06 (1.25, 3.40)	0.004
Demographic, SES, and welfare^d^ ^,^ ^e^	Food insecurity	2.36 (1.77, 3.15)	<0.001	2.23 (1.58, 3.14)	<0.001	3.24 (1.80, 5.86)	<0.001

Abbreviation: OR, odds ratio; CI, confidence interval; SES, socioeconomic status.

^a^ Adjusted for region fixed effects.

^b^ Adjusted for age, marital status, number of household members, self-rated health, chronic disease, smoking, and region fixed effects.

^c^ Adjusted for age, marital status, number of household members, self-rated health, chronic disease, smoking, health insurance, employment status, household income, and region fixed effects.

^d^ Adjusted for age, marital status, number of household members, self-rated health, chronic disease, smoking, health insurance, employment status, household income, free meal services, home-delivered meal services, National Basic Livelihood Security, Basic Pension, and region fixed effects.

^e^ Weighted by inverse probability weights, 1/PS for the food insecure respondents and 1/(1-PS) for the food secure respondents.

Additional analysis explored the moderating role of income support and food assistance programs in the association between food insecurity and suicidal ideation (Table 3). The interaction between food insecurity and free meal services was not associated with suicidal ideation, adjusted for demographic and socioeconomic characteristics (OR, 1.31; 95% CI, 0.72–2.39). The interaction term, food insecurity × home-delivered meal services, was associated with lower odds of suicidal ideation (OR, 0.43; 95% CI, 0.21–0.88). The interaction terms involving National Basic Livelihood Security (OR, 1.02; 95% CI, 0.62–1.70) and Basic Pension (OR, 0.64; 95% CI, 0.10–4.16) were not associated with suicidal ideation.

**TABLE 3:** Association of food insecurity with suicidal ideation conditional on welfare benefits receipt, conditional fixed effects logistic regression results (Korean Welfare Panel Study, South Korea, 2012–2019).

Predictors^a^	OR (95% CI)	*p*-value	OR (95% CI)	*p*-value	OR (95% CI)	*p*-value	OR (95% CI)	*p*-value
Food insecurity	1.68 (1.27, 2.23)	<0.001	2.00 (1.52, 2.63)	<0.001	1.76 (1.25, 2.47)	0.001	2.77 (0.43, 17.8)	0.285
Free meal services	0.72 (0.57, 0.92)	0.008						
Home-delivered meal services			1.60 (1.09, 2.36)	0.017				
National Basic Livelihood Security					0.78 (0.50, 1.21)	0.269		
Basic Pension							2.43 (1.49, 3.95)	<0.001
Food insecurity × free meal	1.31 (0.72, 2.39)	0.381						
Food insecurity × home-delivered meal			0.43 (0.21, 0.88)	0.020				
Food insecurity × National Basic Livelihood Security					1.02 (0.62, 1.70)	0.926		
Food insecurity × Basic Pension							0.64 (0.10, 4.16)	0.638

Abbreviation: OR, odds ratio; CI, confidence interval.

^a^ Adjusted for age, marital status, number of household members, self-rated health, chronic disease, smoking, health insurance, employment status, household income, National Basic Livelihood Security, Basic Pension, free meal services, home-delivered meal services, and region fixed effects.

## Discussion

South Korea has an alarmingly high rate of elderly suicide, with 46.6 suicides occurring for every 100,000 individuals aged 65 and above in 2019 [1]. This rate is much higher than the national average of 26.9 per 100,000 and one of the highest among all OECD countries [1]. The predominant risk factors of this elevated suicide rate include medical conditions (37.5%), economic hardship (28.1%), loneliness and social isolation (15.9%), and family conflicts (11.0%) [24]. Many older adults in Korea face economic difficulties due to insufficient savings and weak social security systems [25, 26]. The high co-payment for medical services further exacerbates their financial hardship [27], making them feel marginalized and stressed about making ends meet.

Food insecurity is an important dimension of economic hardship, given its health and wellbeing implications [9–16]. In this study, we examined the longitudinal association of food insecurity with suicidal ideation among Korean adults aged 65 years and older from the 2012–2019 KoWePS and explored the potential moderating effects of food assistance and income support programs. Three key findings emerged from our analysis. First, becoming food insecure was associated with increased odds of suicidal ideation, even after adjusting for time-varying sociodemographic factors. Second, the link between food insecurity and suicidal ideation was more pronounced in men compared to women. Third, participation in home-delivered meal services mitigated the association between food insecurity and suicidal ideation. Other food assistance and income support programs did not modify the estimated associations. Suicidal ideation is an important precursor of attempted and completed suicide, and thus is a high-priority issue requiring preventive interventions. Our findings underscore the need for more concerted efforts to address food insecurity issue in Korea to help reduce suicide rates among the elderly.

Our findings corroborate the positive association between food insecurity and suicidal outcomes in older adults, as documented in the cross-sectional studies [3, 17, 18]. They also generally align with the psychiatry literature, which demonstrates that food insecurity is a significant predictor of poor mental health, including anxiety and depression [11–16]. The longitudinal design of this study and the focus on Korean sample enables us to offer the first evidence that becoming food insecure is associated with the onset of suicidal thoughts over time in the elderly population in Korea. While material deprivation manifests in various forms at older ages, our research suggests that even moderate food hardship is related to suicidal ideation above and beyond the influence of other competing needs. That food insecurity is linked to suicidal ideation independently of economic conditions indicates a heightened vulnerability to suicide among food insecure individuals that is otherwise not captured by objective hardship indicators [28]. Food insecurity is inherently distinct from material hardship in terms of its mental health effects, necessitating different interventions compared to addressing poverty alone.

There are several possible explanations for the link between food insecurity and increased risk of suicidal thoughts among older adults. One is chronic stress and anxiety due to constant worries of not having enough money to obtain food and maintain a balanced diet [12, 15, 28, 29]. The inability to provide food for oneself or one’s family can lead to feelings of powerlessness, shame, guilt, and hopelessness, negatively impacting their self-esteem and sense of self-worth [30, 31]. These adverse emotions contribute to the development of affective disorders [32] and increase the likelihood of becoming depressed in later years [29]. Moreover, chronic stress can result in maladaptive responses, including impairments in thought processes, concentration, and decision-making [33]. People exhibiting maladaptive responses often engage in self-harm as a means of temporary relief from stressful life events [34]. Another mechanism concerns the mental health effects of poor nutrition and inadequate dietary intake. Food insecurity has been linked to inadequate intakes of micronutrients, such as vitamin B12, zinc, iron, and omega-3 fatty acids [35, 36]. Deficiencies of these nutrients could impair brain functioning and emotion modulation, which is critical for maintaining mental health [37, 38].

The moderating effects of home-delivered meal services further highlight the need for effective interventions to address food insecurity in older adults. A systematic review by Gualtieri et al. [39] shows that home-delivered meal services, such as “Meals on Wheels,” have improved the dietary intake and nutritional status of low-income older adults in the United States. The benefits of home-delivered meal services extend beyond nutrition, relieving stress and anxiety associated with food access and fostering a sense of self-worth and self-esteem [40]. Studies report that interactions with meal delivery volunteers alleviate feelings of social isolation and loneliness among beneficiaries [41, 42]. The meal delivery volunteers often give additional support, helping vulnerable individuals navigate their daily challenges and maintain independence at home [43]. Our findings add to this literature by showing that home delivered meals may improve mental health of food-insecure beneficiaries in Korea.

The insignificant effect of the congregate meal program warrants further research on stigmatizing experiences with the current food assistance interventions. The congregate meal program requires food insecure persons to physically visit the dining hall and line up for food distribution, which could be potentially stigmatizing. Studies of SNAP showed that receiving food assistance was independently associated with greater depressive symptoms [44] and higher odds of suicide ideation, planning, and attempt in the US [45]. One of the reasons that home-delivered meal services, but not congregate meal program, moderates the effect of food insecurity may be because food delivery protects participants’ identity and helps avoid unwanted contact with other beneficiaries. A potential avenue for future research is to identify various features of food assistance programs that can prevent stigmatizing experiences.

### Strengths and Limitations

This study has several limitations. First, the study used self-reported data on suicidal ideation, which could be under-reported due to social desirability bias. It is possible that the responses to the survey question were biased towards a socially desirable one and resulted in an underestimated prevalence of suicidal ideation in the study sample. Second, this study could not examine suicide behaviors. While the KoWePS included questions regarding suicide planning and attempt, there was a lack of enough affirmative responses to construct behavioral indicators. Third, our measure of suicidal ideation was constructed from a single question. Future research could employ a multidimensional approach to measuring suicidal ideation to corroborate our findings. Lastly, this study was unable to empirically assess potential pathways connecting food insecurity to suicidal ideation. Identifying the mechanisms and clarifying their individual roles would enhance our understanding of how food insecurity contributes to suicide and inform the development of intervention programs.

Despite these limitations, this study adds to the literature by confirming the longitudinal association between food insecurity and suicidal ideation among older adults, using a validated measure of food insecurity, and using IPW methods to balance out observed covariate heterogeneity between food secure and insecure respondents. Furthermore, we present the novel finding that home-delivered meal services may protect older persons against the adverse mental health effects of food insecurity.

### Conclusion

The finding that seniors with food insecurity were nearly two times more likely to contemplate suicide than the food secure seniors, and that food delivery program cushions this negative effect of food insecurity point to the importance of addressing senior hunger to reduce suicide. Intervention programs providing food assistance should consider various aspects of delivery to minimize unintended consequences while maximizing potential benefits.
