# Supplementary material for: Longitudinal Associations Between Food Insecurity and Suicidal Ideation Among Adults Aged ≥65 in the Korean Welfare Panel Study
Source: Int J Public Health. 2023 Jun 5;68:1605618. doi: 10.3389/ijph.2023.1605618 (PMC10277513; doi:10.3389/ijph.2023.1605618)
Supplement: Supplementary file 1 [file DataSheet1.PDF]

Supplementary Material

TABLE S1 Household Food Security Survey Module in the Korean Welfare Panel Study

Instructions: in the preceding year, have your household had the following experiences regarding food and diet?

1. In the preceding year, because of economic hardship, I did not have enough money to buy food even when I was out of food.
   □ often □ sometimes □ never □ don't know or refusal
2. In the preceding year, because of economic hardship, I did not have enough money to have balanced meals (in sufficient amounts of various diets).
   □ often □ sometimes □ never □ don't know or refusal
3. In the preceding year, have any adults in your household reduced the amount of meals or skipped meals because there was not enough money to buy food?
   □ yes □ no □ don't know or refusal
   (If answered yes) How often did this happen?
   □ almost every month □ some months (but not every month) □ 1 or 2 months
4. In the preceding year, have you eaten less than you felt you should because there was not enough money to buy food?
   □ yes □ no □ don't know or refusal
5. In the preceding year, have you been unable to eat even when you were hungry because there was not enough money to buy food?
   □ yes □ no □ don't know or refusal

Note: translated by author.
